# Investigating effect of climate warming on the population declines of *Sympetrum frequens* during the 1990s in three regions in Japan

**DOI:** 10.1038/s41598-020-69532-8

**Published:** 2020-07-29

**Authors:** Kosuke Nakanishi, Dai Koide, Hiroyuki Yokomizo, Taku Kadoya, Takehiko I. Hayashi

**Affiliations:** National Institute for Environmental Studies, Onogawa 16-2, Tsukuba, Ibaraki 305-8506 Japan

**Keywords:** Climate-change ecology, Conservation biology

## Abstract

Climate warming is of concern as a key factor in the worldwide decline in insect populations. In Japan, numbers of a common dragonfly in rice paddy fields, *Sympetrum frequens*, decreased sharply in the 1990s. Because *S. frequens* migrates to cooler mountains in summer, climate warming has been suggested as one of the main causes of the population decline in addition to agronomic factors. Here, we analysed the relation between summer temperatures and population densities of *S. frequens* and the related *S. infuscatum*, which does not migrate to mountains in summer, using published population monitoring data and temperature data from three regions (Toyama, Ishikawa, and Shizuoka) in Japan. Decadal differences in summer temperatures lay within the range of fluctuations among years, suggesting that an increase in summer temperatures cannot explain the past sharp population declines. However, regression analyses using monitoring data from Toyama showed that the population dynamics of both species in autumn are negatively correlated with summer temperatures in the same year. These results suggest that high temperatures in summer directly affect adult mortality to an extent that results in a decrease in population growth.

## Introduction

Insect abundance and biodiversity have been declining worldwide over the last few decades^1,2^. Climate warming is of concern as one of the key factors in the declines, in addition to habitat loss, pollution, and invasive species^1,3–5^. Because ambient temperature directly affects insect development, survival, and fecundity^6–9^, climate warming can affect insects’ population growth and distribution. Climate warming can also affect insects indirectly via biological interactions with other species, such as competition, predation, herbivory, and parasitism^10,11^. Many insects thus have gone locally extinct and shifted their ranges to higher latitudes and altitudes with climate warming^12–14^. Dragonflies (Odonata) are regarded as a suitable indicator group for climate change for several reasons: their developmental rate is strongly correlated with temperature; their distribution is limited by temperature; they are migratory carnivores that play important roles as intermediate predators in both aquatic and terrestrial ecosystems; and they have a long history of scientific research providing rich data^15–17^. Many dragonflies have shifted their range polewards over the last several decades following the loss of thermally suitable habitat^12,18,19^.

Japan has a highly rich dragonfly fauna compared with other countries^20^, with 203 resident species recorded^21^. In recent decades, however, many dragonfly species in Japan have faced extinction through habitat degradation^22^. *Sympetrum frequens* (Odonata: Libellulidae), known familiarly as the “red dragonfly”, is one of the most common dragonfly species in Japan^23^ and is symbolic of the Japanese countryside^24^. It commonly reproduces in rice paddy fields, which occupy ~ 24,000 km^2^ (much larger than the natural wetland area of 820 km^2^) in Japan^25,26^, and is an important predator in agricultural ecosystems in both nymphal aquatic and adult terrestrial stages. During the 1990s, its numbers decreased sharply to near extinction (to 1/100th of their levels in the early 1990s) in many regions of Japan^27–29^. The application of systemic insecticides (e.g., the phenylpyrazole fipronil and the neonicotinoid imidacloprid) to rice fields—the main breeding sites of the species—is suspected as the main cause of the decline^30,31^. Climate warming is held as another potential cause^30,32^. Among dragonfly species in Japan, *S. frequens* is thought to be highly vulnerable to climate warming because of its unique life history: during summer (July–August), the immature adults migrate to cooler mountainous areas often tens of kilometres from their natal habitats, warmer lowland rice fields, and the matured adults return to the fields in autumn^33^.

There are some hypothetical interpretations for the migratory behaviour of *S. frequens*. Interpretation 1 is that the behaviour is an adaptation of the ancestors of *S. frequens* (a race of the continental species *S. depressiusculum*) to the warmer climate in Japan after the last glacial period^34^. Because they inhabited a cooler climate and had no migratory behaviour, in a warmer climate they needed to seek cooler areas to avoid the effects of heat stress such as increased mortality and decreased reproductive performance. Immature adults, in particular, are thought to be more vulnerable to high temperatures than mature adults because their cuticle is incompletely hardened and therefore more prone to water loss^35^. Although there are no data on threshold temperatures of heat stress in *S. frequens*, in many insect species heat stress starts within a temperature range of 28–32 °C^36^. In lowland areas, the daily mean temperature in summer can often reach that range throughout Japan. Interpretation 2 is that the behaviour allows a pre-reproductive period (i.e., reproductive diapause), which results in overwintering in the egg stage^37^. If adults initiate mating and egg laying without reproductive diapause, the eggs could hatch in autumn, leaving the early nymphs unable to survive in winter^37^. On the basis of its distributional record and mean temperature data, Uéda^37^ suggested that this species needs a summer habitat where the mean temperature is below 23 °C to permit reproductive diapause. In many mountainous areas of Japan, where *S. frequens* is observed in summer, the mean summer temperature does not exceed 23 °C. For these reasons, population dynamics of *S. frequens* are expected to be highly affected by summer temperatures. However, no previous study has examined the relation between temperature and population dynamics of *S. frequens*.

Here, we aimed at answering whether climate warming can explain the sharp decline in numbers of *S. frequens* in the 1990s. We also examined whether an increase in summer temperature generally is associated with population growth of *Sympetrum* dragonflies. We also targeted *S. infuscatum*, another common dragonfly reproducing in rice fields throughout Japan^38^, whose numbers also decreased in the 1990s^27^. In contrast to *S. frequens*, *S. infuscatum* does not migrate to distant cooler mountainous areas; instead, after emergence, the adults migrate to forest gaps near rice fields^39^. We examined whether this difference in their life history is related to vulnerability to high summer temperatures.

## Methods

### Dragonfly population data

We used published population monitoring data of the two *Sympetrum* species collected in Toyama^27^, Ishikawa^28^, and Shizuoka^29^ prefectures. Census methods differed among prefectures. In Toyama, matured adults of the two species were counted for a few tens of minutes at several hundred locations within a broad range of the prefecture during October in every year from 1993 to 2011^27^. The data gave the number of individuals per hour within the prefecture in a month. In Ishikawa^28^ and Shizuoka^29^, immature adults of *S. frequens* were counted at a single site in August in several years from 1989 to 2010 and from 1993 to 2009, respectively. Because the two species have a univoltine life cycle^21,33^, the individuals observed belong to populations emerged in the same year (June–July). The data gave the number of individuals per 100 m and per hour per surveyor, respectively. To examine the association between long-term trends of summer temperature and population dynamics of the two species during the 1990s, we used the population data of these three prefectures. In regression analyses examining the relation between summer temperature and population dynamics, we used only the Toyama data, which cover 19 years, because the population data in the other prefectures were not continuous. We then used values of a parameter estimated from a regression model to project the population dynamics of *S. frequens* in Toyama.

### Temperature data calculations

As an index of summer temperature, we used the 90th percentile values of the daily mean temperature (*TEMP*) during July–August, the hottest period in Japan. Because the ancestors of *S. frequens* inhabited a cooler continental climate^34^, we assumed that *S. frequens* is likely to suffer heat stress more seriously as the temperature increases, as do many other insects^36^. We used the 90th percentile as the upper bound because the seasonal upper temperature is expected to be a more appropriate indicator associated with annual population growth. In addition, we used the daily mean rather than the daily maximum temperature in summer as an index of direct high-temperature damage to adult dragonflies, because a high mean reflects a longer duration of high temperature, which can cause greater heat stress in adult dragonflies, than a high momentary value. Past temperature data from a ~ 1-km^2^ grid were obtained from NARO Agro-Meteorological Grid Square Data (AMGSD)^40^, a set of spatially interpolated data calculated from values measured by the Automated Meteorological Data Acquisition System by the Japan Meteorological Agency^41^. We calculated spatial mean values of the 90th percentile temperature of the squares in each prefecture from 1981 to 2017. We considered it reasonable to analyse the relation between spatial mean temperature within a prefecture and abundance of both migratory *S. frequens* and non-migratory *S. infuscatum* for two reasons, both based on the fact that prefectural borders are often formed by mountain ridges. First, temperatures at different altitude (e.g., lowland and mountain) within a prefecture have a linear relationship with each other. Second, *S. frequens* appears to complete its life cycle mostly within a prefecture (i.e., matured adults stay in the mountains in summer and later return to their natal area)^32^. For these reasons, because we used Δ*TEMP* (i.e., annual difference, not absolute value) as an index of temperature, we expected Δ*TEMP* of spatial mean values in a prefecture to correlate with those values of each species’ range.

To qualitatively analyse time trends of *TEMP* during the period of the sharp decline of *S. frequens* (i.e., from 1990 to 1999), we calculated the 10-year difference (*DIFF*), rate of change (*RATE*), and standardized difference (*STDIFF*) of *TEMP* in each prefecture during each decade of the 1980s, 1990s, and 2000s. Because annual *TEMP* fluctuated too widely to properly represent the decadal difference, we used a 5-year moving average to reduce variabilities among individual years and see long-term time trends^42–44^. We calculated the difference (*DIFF*_*i*,1990s_), percentage rate of change (*RATE*_*i*,1990s_), and standardized difference (*STDIFF*_*i*,1990s_) in prefecture *i* in the 1990s as:

$$\begin{aligned} & DIFF_{i,1990s} = TEMP_{i,1999MA} {-}TEMP_{i,1990MA} \\ & RATE_{i,1990s} = \, \left\{ {\left( {TEMP_{i,1999MA} {-}TEMP_{i,1990MA} } \right) \, /TEMP_{i,1990MA} } \right\} \, \times \, 100 \\ & STDIFF_{i,1990s} = DIFF_{i,1990s} /SD_{i,1990s} \\ \end{aligned}$$

where *TEMP*_*i*,1999MA_ and *TEMP*_*i*,1990MA_ are *TEMP* of the 5-year moving average (MA, 5-year mean between years *t* − 2 and *t* + 2 in year *t*) in prefecture *i* in 1999 and 1990, respectively; *SD*_*i*,1990s_ is the standard deviation of the annual values of *TEMP* in prefecture *i* in the 1990s; and *STDIFF* represents the long-term difference standardized to the magnitude of short-term (i.e., year-by-year) variation. We calculated these index values for each decade. Note that the starting point of the 1980s was 1983 owing to the limited availability of dragonfly data.

### Regression analyses

We examined the relations between the annual difference in *TEMP* (∆*TEMP*) and population growth rates of the two *Sympetrum* species in Toyama. We used ∆*TEMP* rather than absolute *TEMP* as a variable for reducing the temporal autocorrelation over years in the models. Our supplementary analyses showed that the models using absolute *TEMP* had no substantial difference in the main results of this study from models using ∆*TEMP* (see Supplementary Note S1). We assumed that the relationship between ∆*TEMP* and population growth can be approximated by a linear model because the range of ∆*TEMP* in the period was not too large to reject a linear approximation. We based two statistical models on the two interpretations (see “Introduction” section) of the migratory behaviour of *S. frequens*.

In interpretation 1 (the migratory behaviour avoids high temperatures in summer as an adaptation to a warmer climate^34^), an increase in *TEMP* will increase adult mortality owing to heat stress. This implies a negative relation between *TEMP* and the abundance of a dragonfly within the same year. We constructed the following statistical model:

$$\lambda_{t} = {\ln}N_{t} {-}{\ln}N_{{t - {1}}} = \, \alpha + \beta \Delta TEMP_{t} + \, \varepsilon_{t} , \qquad \text{(Model 1)}$$

where λ_*t*_ is the annual population growth rate of a dragonfly in year *t*; *N*_*t*_ (*N*_*t*−1_) is a population density index (number of individuals/h) in year *t* (year *t − *1) recorded in October in Toyama^27^; α is the intercept; ∆*TEMP*_*t*_ is the difference in *TEMP* (°C) between year *t* and year *t − *1 (∆*TEMP*_*t*_ = *TEMP*_*t*_ − *TEMP*_*t*−1_); β is the coefficient; and ε_*t*_ is the error term in year *t*. This model implies that the same *TEMP* in year *t* and year *t − *1 (i.e., ∆*TEMP*_*t*_ = 0) leads to a zero growth rate when effects of other factors are negligible. We assumed that values of ε_*t*_ were independent between years; that is, temporal autocorrelations over years do not exist or are properly modelled in the regressions. This assumption was statistically tested by the Durbin–Watson test.

In interpretation 2 (the migratory behaviour allows *S. frequens* to overwinter in the egg stage^37^), an increase in *TEMP* will promote earlier reproduction (i.e., disturb reproductive diapause) and increase mortality of early-emerged nymphs in winter owing to drying or low temperature. Therefore, an increase in *TEMP* should be related to the adult density in the following year. We constructed the following statistical model:

$$\lambda_{t} = {\ln}N_{t} {-}{\ln}N_{{t - {1}}} = \, \alpha + \beta \Delta TEMP_{{t - {1}}} + \, \varepsilon_{t} , \qquad \text{(Model 2)}$$

where ∆*TEMP*_*t*−1_ is the difference in *TEMP* between year *t* − 1 and year *t* − 2 (∆*TEMP*_*t*−1_ = *TEMP*_*t*−1_ − *TEMP*_*t*−2_).

We conducted linear regression analyses of Models 1 and 2 with both species to examine the relations between *TEMP* and density. Because the population density had nearly bottomed by 2005 in Toyama and the subsequent data are likely to consistently bias the growth rate towards an asymmetrical (i.e., increasing) trend owing to the lower bound of the density, we used only the data between 1993 and 2004 in the analyses for both species. We used R v. 3.6.1^45^ software for the analyses, and the *lmtest* package^46^ for the Durbin–Watson test. Data and R code are available in the Supplementary Materials online.

In the above models, the effects of other environmental factors that are independent of ∆*TEMP* are assumed to be included in the error term ε. If these factors are independent of ∆*TEMP*, their values will not statistically affect the consistent estimator of the regression coefficient of ∆*TEMP*. For example, many agronomic factors may affect growth rate but are expected to be independent of ∆*TEMP* (though not absolute temperature). Some other potentially non-independent environmental factors (e.g., moisture levels and UV radiation) could affect growth rate. However, because previous studies suggest that these effects were much smaller than the direct effects of temperature^9^, we assumed that they did not have substantial influence on the consistent estimator for ∆*TEMP*. Among other environmental factors, insecticide application to rice fields can be a major cause of population declines of *S. frequens*^30,31^. In a supplementary analysis (Supplementary Note S2), we tested the possible effects of this important factor on the estimates of the effect of ∆*TEMP* by analysing a model that added insecticide use as a covariate to the above models, using insecticide use data in Toyama Prefecture^30^. This analysis revealed that insecticide use had no substantial influence on the results of this study.

### Projection of population densities by using regression parameter

We projected the population density of *S. frequens* in Toyama by using the value of β of the above models under the assumption that only temperature affects population dynamics. Note that the aim of this projection was to test whether the effect of temperature by itself can substantially explain the population dynamics and not to simulate realistic population dynamics by using models with various environmental parameters.

Because Model 1 performed better than Model 2 (see results of regression analyses in “Results” section), we used the β of Model 1 in the projections and assumed that *TEMP* directly affects the population density of *S. frequens* within the same year. We treated the intercept (α, a constant time trend independent of temperature) and error term (ε_*t*_) as 0 in the model, and calculated the annual population growth rate of *S. frequens* (λ_*t*_) in year *t* with β as:

$$\lambda_{t} = {\ln}N_{t} {-}{\ln}N_{{t - {1}}} = \, \beta \Delta TEMP_{t} ,$$

where *N*_*t*_ is population density in year *t*, and ∆*TEMP*_*t*_ is the difference in *TEMP* between year *t* and year *t* − 1. Note that this calculation provides a theoretical projection of population dynamics under an assumption that only temperature affects population density. For past population dynamics, we calculated population density during *S. frequens* observation period in Toyama (i.e., 1993–2011)^27^ by using the temperature data from AMGSD. We set the population density of the first year of the observation (i.e., 1993) at 1, and calculated abundance relative to the initial value in Toyama in subsequent years.

## Results

### Decadal time trends of temperature compared with population dynamics

**Figure 1:**
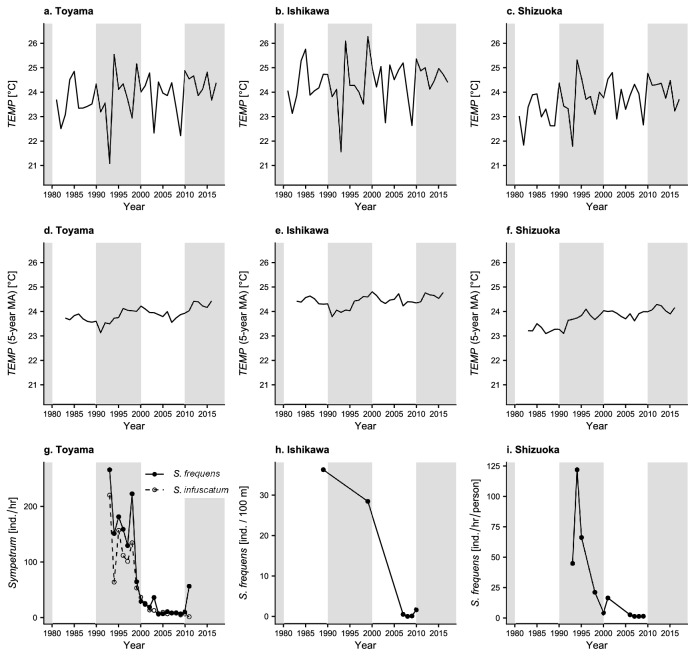
Changes in (**a**–**c**) the annual 90th percentile values of daily mean temperature during July–August (*TEMP*) and (**d**–**f**) their 5-year moving averages (MA), and (**g**–**i**) abundances of *Sympetrum* dragonflies in the three prefectures. Dragonfly data come from Futahashi (2012) for Toyama, Uéda (2012) for Ishikawa, and Fukui (2012) for Shizuoka.

The annual *TEMP* fluctuated widely in all prefectures (Fig. 1a–c). The 5-year moving average showed a slightly increasing trend between the 1980s and the 2010s in all three prefectures (Fig. 1d–f). In particular, *TEMP* increased (by 1.19–2.43%) in the 1990s in all prefectures (Table 1). However, as all values of *DIFF* were < 1 SD during the period (the absolute values of *STDIFF* ranged from 0.22 to 0.59 °C; Table 1), the size of the 10-year difference in *TEMP* lay within the range of the fluctuations among years. The population density of *S. frequens* decreased by as much as 97.4% from 1993 to 2004 in Toyama (Fig. 1g), whereas the standardized increase in *TEMP* in the 1990s (i.e., *STDIFF*_*i*,1990s_) was only 0.33 °C (Table 1).

**Table 1:** Ten-year difference (*DIFF*) in and rate of change (*RATE*) of the 90th percentile values of daily mean temperature during July–August (*TEMP*) during each decade and standardized difference (*STDIFF*) of the annual *TEMP* during 3 decades in the three prefectures.

	Toyama			Ishikawa			Shizuoka		
	1980s^a^	1990s	2000s	1980s^a^	1990s	2000s	1980s^a^	1990s	2000s
*DIFF* (°C)	− 0.17	0.41	− 0.35	− 0.12	0.29	− 0.42	0.06	0.56	− 0.04
*RATE* (%)	− 0.71	1.73	− 1.46	− 0.49	1.19	− 1.69	0.25	2.43	− 0.18
*STDIFF* (°C)	− 0.25	0.33	− 0.41	− 0.16	0.22	− 0.44	0.11	0.59	− 0.06

^a^From 1983 to 1989.

Although the annual patterns of *TEMP* were similar among prefectures, the sharp population declines of *S. frequens* started at different times and were not coincident among the prefectures (Fig. 1g–i): between the mid and late 1990s in Toyama and Shizuoka but in the early 2000s in Ishikawa (Fig. 1g–i). This difference indicates that the sharp population declines and the fluctuations of *TEMP* were not always coincident. In addition, *S. infuscatum*, which does not migrate to high mountainous areas, decreased as sharply as *S. frequens* in Toyama^27^ (Fig. 1g).

### Association between temperature and dragonflies

**Figure 2:**
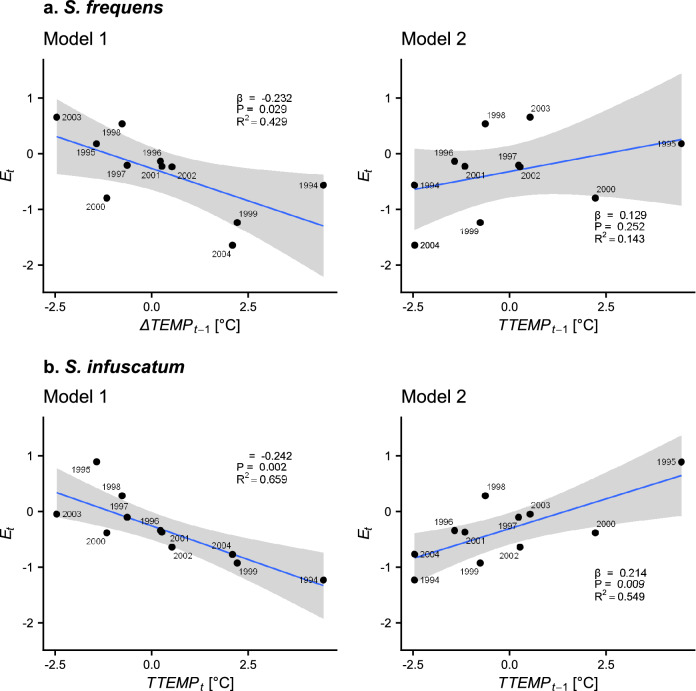
Association between ∆*TEMP* in the 90th percentile values of daily mean temperature during July–August (∆*TEMP*_*t*_) and population growth rate (λ_*t*_) of (**a**) *Sympetrum frequens* and (**b**) *S. infuscatum* in Toyama Prefecture from 1993 to 2004. The shaded zone represents the 95% confidence interval. The labels indicate year *t*. The results of regression analyses are shown in each panel.

∆*TEMP*_*t*_ was negatively correlated with the population growth of *S. frequens* (λ_*t*_) in the same year (Fig. 2a: Model 1, β = − 0.232, *P* = 0.029, *R*^2^ = 0.429, Akaike’s Information Criteria [AIC] = 22.07). AIC for the null model (i.e., the model without ∆*TEMP*_*t*_) was 26.24; this shows that ∆*TEMP*_*t*_ has non-negligible information for predicting population growth. We did not find a significant correlation between ∆*TEMP* in the previous year (∆*TEMP*_*t*−1_) and the population growth of *S. frequens* in the succeeding year (λ_*t*_) (Fig. 2a: Model 2, *R*^2^ = 0.143, AIC = 26.54). On the other hand, ∆*TEMP* in both the same year (∆*TEMP*_*t*_) and the previous year (∆*TEMP*_*t*−1_) was significantly correlated with the population growth of *S. infuscatum* (λ_*t*_) (Fig. 2b: Model 1, β = − 0.242, *P* = 0.002, *R*^2^ = 0.659, AIC = 12.63, AIC for null model = 22.45; Model 2, β = 0.214, *P* = 0.009, *R*^2^ = 0.549, AIC = 15.70). According to the coefficient of determination (*R*^2^) and AIC, Model 1 performed better than Model 2 for *S. infuscatum*. The Durbin–Watson test (see Supplementary code) showed that our regression models had no temporal autocorrelation.

### Projection of past abundance

**Figure 3:**
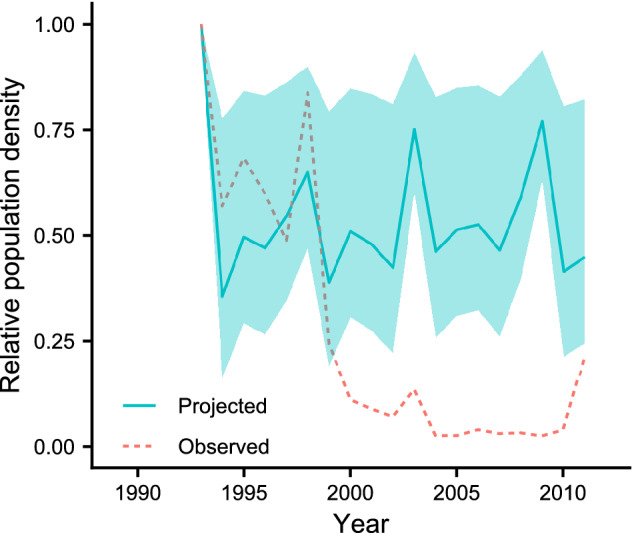
Relative population densities of *Sympetrum frequens* projected by using the estimated coefficient of the regression analysis (Model 1) and the temperature data of AMGSD (—), and the observed relative abundance (- - -) in Toyama Prefecture. The shaded zone represents the range of projected relative population densities calculated by using the upper and lower limits of the 95% confidence interval of the estimated coefficients. The observed data come from Futahashi (2012).

The projection did not reproduce the sharp decline observed in the 2000s in Toyama in the relative abundance of *S. frequens* (Fig. 3) or *S. infuscatum* (Fig. S1). Thus, the severe decline in the 1990s cannot be explained by temperature alone.

## Discussion

The comparisons between 10-year time trends of *TEMP* and population densities showed that climate warming was not the main cause of the sharp declines in numbers of *S. frequens* in the 1990s for three reasons. First, the size of the 10-year differences in *TEMP* lay within the range of its fluctuations among years (Table 1), although *TEMP* increased slightly in all prefectures in the 1990s when numbers decreased sharply (Fig. 1). Second, the timings of the sharp declines were not coincident among the prefectures, whereas the annual fluctuations of *TEMP* were similar (Fig. 1). Third, our simple projection of the past population dynamics of *S. frequens* shows that temperature was not enough to explain the past population dynamics (Fig. 3). These results are consistent with a previous argument that the systemic insecticides applied to rice fields (particularly fipronil) introduced in the 1990s, in addition to other agronomic factors (e.g., midsummer drainage and crop rotation), were the main cause of the sharp decline of *S. frequens* populations in Japan^30,31^.

However, the regression analyses suggest that population dynamics of the two species are affected to an extent by summer temperature. The difference in *TEMP* in the same year was significantly negatively correlated with the population growth of *S. frequens* (Model 1), but the difference in *TEMP* in the previous year was not (Model 2; Fig. 2a). These results indicate that high temperatures in summer directly affected adult mortality, not reproductive success (i.e., population in the succeeding year), which supports interpretation 1: that the migratory behaviour of *S. frequens* avoids direct high-temperature damage in summer^34^. There may also be indirect negative effects of high temperatures on mortality via interaction with other species (e.g., competition, predation, and parasitism)^10,11^.

We expected that the vulnerabilities of the two *Sympetrum* species to high summer temperatures differed because of the difference in their migratory behaviour in summer. However, there was no clear difference in the magnitude of the estimated coefficient (β) (Fig. 2). This result suggests that migration of *S. frequens* to cooler mountainous areas is not very effective at avoiding a decrease in population growth owing to direct high-temperature damage in summer. Both species might be affected by climate warming to similar degrees.

The estimated β coefficients of Models 1 and 2 of *S. infuscatum* (Fig. 2b) and *S. frequens* (Fig. 2a) had opposite signs: ∆*TEMP* was negatively correlated with population growth in the same year but positively correlated with it in the succeeding year. These results are explained by the fact that both ∆*TEMP*_*t*_ (= *TEMP*_*t*_ – *TEMP*_*t*−1_) and ∆*TEMP*_*t*−1_ (= *TEMP*_*t*−1_ – *TEMP*_*t*−2_) use the same term, *TEMP*_*t*−1_. For example, when *TEMP*_*t*−1_ increases, *TEMP*_*t*−1_ can increase, while ∆*TEMP*_*t*_ can decrease. Because Model 1 was the better predictor as judged by *R*^2^ and AIC values, it is straightforward to consider that high temperatures directly increase dragonfly mortality in the same year.

In supplementary analyses, we tested the associations between the population growth of *S. frequens* and its summer habitat area (SHA, km^2^), where the mean temperature during July–August does not exceed 23 °C^37^, in the same way as *TEMP* (Supplementary Note S3). We also tested the correlation with ∆SHA calculated under temperature thresholds of 21–25 °C to test the validity of the 23 °C threshold. There were significant positive correlations between ∆SHA calculated under all thresholds and population growth of *S. frequens* in the same year (Fig. S2) but not in the previous year (Fig. S3). The model using SHA calculated at a threshold of 25 °C was the best model (Fig. S2e). Although SHA may also be a key factor affecting population growth, it does not change the main results of this study. Because there was a fairly linear correlation between *TEMP* and ∆SHA at the prefecture scale, we need to examine the mechanism of the effect of summer temperature on the population dynamics of *S. frequens* by considering their temporospatial migratory habit in summer at a more local scale. Also, we need to reconsider the definition of summer habitat by Uéda^37^.

There is little available, statistically analysable, long-term, wide-scale population data on dragonfly populations. We could analyse population data from only three regions and conduct regression analyses using data from only one of them. Because the vulnerability of *S. frequens* to summer temperatures should not differ much among regions in Japan, our results suggest that the sharp population declines of *S. frequens* in various regions in Japan were not caused by climate warming. On the other hand, our results suggest that high temperatures in summer could still affect population growth to an extent. In the 2090s, the decadal mean value of *TEMP* in Toyama is expected to rise to 28.0 °C, 3.8 °C higher than that in the 2020s under the RCP 8.5 scenario of the global climate model (see Supplementary Note S4 and Fig. S4). In the supplementary material, we provide a “what if” simulation based on the assumption that only temperature can affect population dynamics under future climate change (Figs. S4, S5). The results suggest that temperature could drive the decline of dragonfly populations in the long term. Monitoring of dragonfly populations in various localities and statistical analyses with consideration of other environmental factors corresponding to habitat range will be necessary for quantifying the effects of temperature and for devising strategies for the conservation of dragonflies.

## Supplementary information

Supplementary Information 1.

Supplementary Information 2.

Supplementary Information 3.
