# Author Correction: Investigating effect of climate warming on the population declines of *Sympetrum frequens* during the 1990s in three regions in Japan

**DOI:** 10.1038/s41598-020-78513-w

**Published:** 2020-12-07

**Authors:** Kosuke Nakanishi, Dai Koide, Hiroyuki Yokomizo, Taku Kadoya, Takehiko I. Hayashi

**Affiliations:** National Institute for Environmental Studies, Onogawa 16‑2, Tsukuba, Ibaraki 305‑8506 Japan

Correction to: *Scientific Reports* 10.1038/s41598-020-69532-8, published online 29 July 2020

This Article contains error in some characters in Figure 2. The correct Figure 2 appears below as Figure 2.

**Figure 2:**
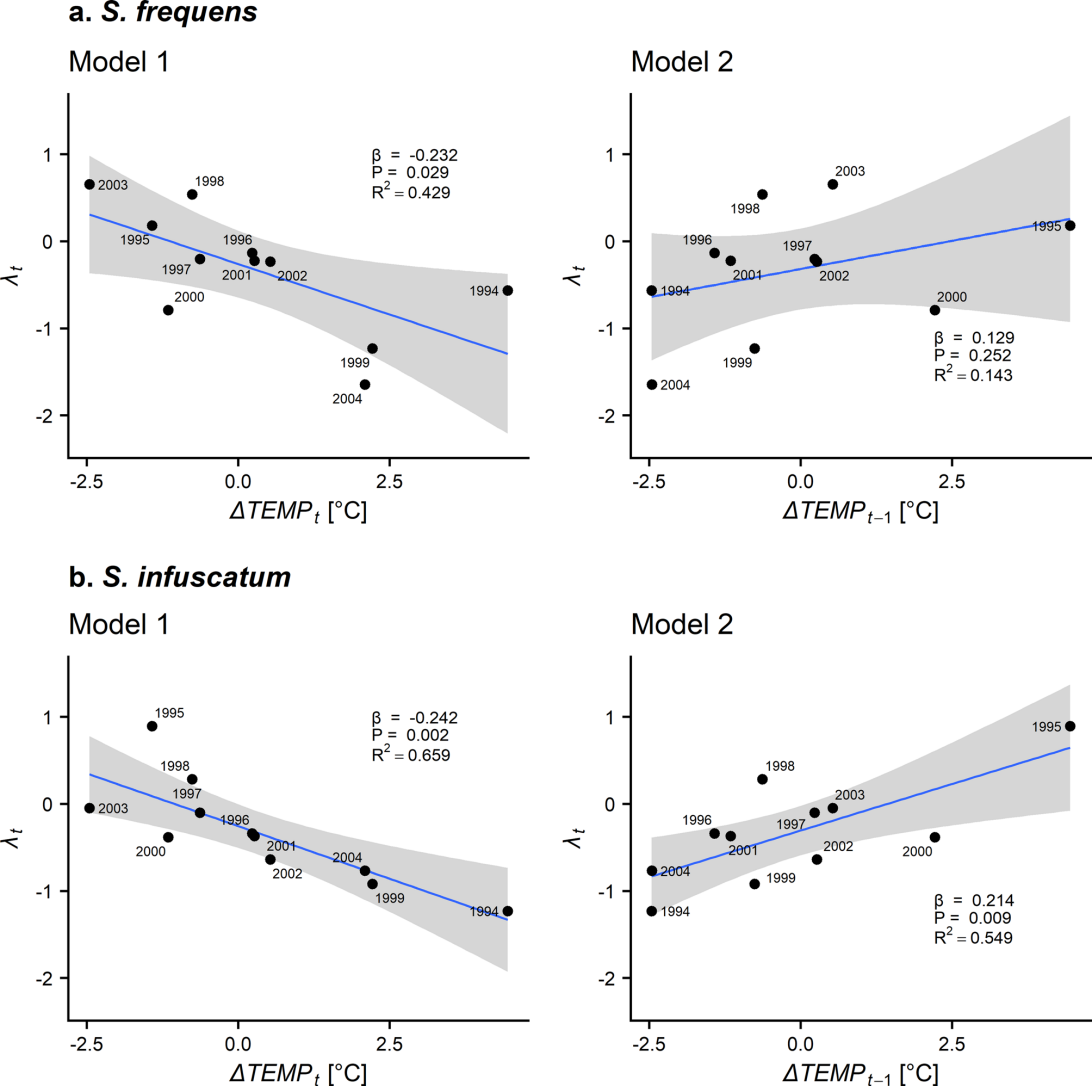
Association between Δ*TEMP* in the 90th percentile values of daily mean temperature during July–August (Δ*TEMP*_*t*_) and population growth rate (λ_*t*_) of (**a**) *Sympetrum frequens* and (**b**)* S. infuscatum* in Toyama Prefecture from 1993 to 2004. The shaded zone represents the 95% confidence interval. The labels indicate year *t*. The results of regression analyses are shown in each panel.

