# Supplementary material for: Investigating effect of climate warming on the population declines of Sympetrum frequens during the 1990s in three regions in Japan
Source: Sci Rep. 2020 Jul 29;10:12719. doi: 10.1038/s41598-020-69532-8 (PMC7391746; doi:10.1038/s41598-020-69532-8)
Supplement: Supplementary file 1 — Supplementary Information 1. [file 41598_2020_69532_MOESM1_ESM.docx]

# Supplementary Information

**Title:** Investigating effect of climate warming on the population declines of *Sympetrum frequens* during the 1990s in three regions in Japan

**Authors:** Kosuke Nakanishi^*^, Dai Koide, Hiroyuki Yokomizo, Taku Kadoya, Takehiko I. Hayashi

National Institute for Environmental Studies, Onogawa 16-2, Tsukuba, Ibaraki 305-8506, Japan

*Corresponding author: nakanishi.kosuke@nies.go.jp

# Note S1. Comparison between temperature difference and absolute temperature as a variable in the regression analyses

## S1.1. Methods

To examine the validity of using the annual difference in temperature (∆*TEMP*), not the absolute value (*TEMP*), as a variable in the regression analyses, we analysed the relation between *TEMP* and population growth as in the main analyses (see “Regression analyses” in Methods) with the following models:

λ*_t_* = α + β*TEMP_t_* + ε*_t_*, (Model 1′)

λ*_t_* = α + β*TEMP_t_*_−1_ + ε*_t_*, (Model 2′)

where λ*_t_* is the annual population growth rate of a dragonfly in year *t*, defined as λ*_t_* = ln*N_t_* − ln*N_t_*_−1_; *N_t_* is the population density index (number of individuals/h) in year *t* recorded in October in Toyama (Futahashi, 2012); α is the intercept; *TEMP_t_* (*TEMP_t−1_*) is the 90th percentile value of daily mean temperature during July–August in year *t* (year *t* − 1) (°C); β is the coefficient; and ε*_t_* is the error term in year *t*.

We conducted linear regressions with these models for *Sympetrum frequens* and *S. infuscatum* as in the main analyses (see “Regression analyses” in Methods).

## S1.2. Results and interpretations

In all cases, the results from the two corresponding models were not largely different from the models using ∆*TEMP* (Table S1, S2). Thus, the selection of the temperature value (∆*TEMP* or *TEMP*) as a variable had no substantial influence on the results of this study. In addition, the model for *S. frequens* using *TEMP* (Model 1′) had a high possibility of temporal autocorrelation over years (DW = 1.12, *P* = 0.058). This indicates that using ∆*TEMP* is a better choice to avoid potential estimation errors due to autocorrelation.

**Table S1.** Results of regression using temperature difference and absolute temperature as a variable for examining population growth of *Sympetrum frequens*.

|  | Explanatory variable | Coefficient  (estimate ± SE) | *P*-value | AIC | DW test^†^ |
| --- | --- | --- | --- | --- | --- |
| Model 1 | ∆*TEMP_t_* | −0.232 ± 0.089 | 0.029* | 21.07 | DW = 1.67, *P* = 0.308 |
| Model 1′ | *TEMP_t_* | −0.510 ± 0.187 | 0.023* | 21.61 | DW = 1.12, *P* = 0.058 |
| Model 2 | ∆*TEMP_t_*_−1_ | 0.129 ± 0.106 | 0.252 | 26.54 | DW = 2.29, *P* = 0.743 |
| Model 2′ | *TEMP_t_*_−1_ | 0.281 ± 0.154 | 0.101 | 24.77 | DW = 2.12, *P* = 0.587 |

^†^Durbin–Watson test.

**P* < 0.05.

**Table S2.** Results of regression using temperature difference and absolute temperature as a variable for examining population growth of *Sympetrum infuscatum*.

|  | Explanatory variable | Coefficient  (estimate ± SE) | *P*-value | AIC | DW test^†^ |
| --- | --- | --- | --- | --- | --- |
| Model 1 | ∆*TEMP_t_* | −0.242 ± 0.058 | 0.002** | 12.63 | DW = 1.66, *P* = 0.298 |
| Model 1′ | *TEMP_t_* | −0.421 ± 0.160 | 0.028* | 18.20 | DW = 2.01, *P* = 0.546 |
| Model 2 | ∆*TEMP_t_*_−1_ | 0.214 ± 0.065 | 0.0091** | 15.70 | DW = 2.12, *P* = 0.647 |
| Model 2′ | *TEMP_t_*_−1_ | 0.350 ± 0.097 | 0.006** | 14.61 | DW = 1.90, *P* = 0.438 |

^†^Durbin–Watson test.

***P* < 0.01; **P* < 0.05.

# Note S2. Regression analyses including insecticide use

## S2.1. Methods

Insecticide application to rice fields can be a major non-independent factor that can cause population declines of *S. frequens* (Nakanishi et al., 2018, 2020). In a preliminary analysis, we tested a model that added insecticide use as a covariate to the above models by using insecticide use ratios (for nursery box application to rice seedlings) in Toyama Prefecture (Nakanishi et al., 2018). The use ratios represent the proportion of potential reproductive sites exposed to an insecticide. We added the annual difference in insecticide use ratio as a covariate into Model 1 (see “Regression analyses” in Methods) according to the methods of Nakanishi et al. (2020) as:

λ*_t_* = α + β_TEMP__​_∆*TEMP_t_* + β_NNFP​_∆INSE_NNFP,_*_t_* + β_CART​_∆INSE_CART,_*_t_*
 + β_CARB​_∆INSE_CARB,_*_t_* + ε*_t_*, (Model 1″)

where λ*_t_* is the annual population growth rate of a dragonfly in year *t*, defined as λ*_t_* = ln*N_t_* − ln*N_t_*_−1_; *N_t_* is the population density index (number of individuals/h) in year *t* recorded in October in Toyama (Futahashi, 2012); α is the intercept; β is a coefficient; ∆*TEMP_t_* is the difference in *TEMP* (°C) between year *t* and year *t* − 1 (∆*TEMP_t_* = *TEMP_t_* − *TEMP_t_*_−1_); NNFP = neonicotinoids (imidacloprid, dinotefuran, clothianidin, and thiamethoxam) + fipronil, CART = cartap, and CARB = carbosulfan; ∆*INSE*_XXXX_*_,t_* represents the difference in the use ratios of insecticides between years *t* and *t* − 1; and ε*_t_* is the error term in year *t*. (We did not include benfuracarb as a covariate because its use ratios in the target period were small: <5%.)

As an alternative way of aggregating the insecticide use ratios, we used the following model:

λ*_t_* = α + β*_TEMP_*_​_∆*TEMP_t_* + β*_TOTAL_*_​_∆*INSE_TOTAL,t_* + ε*_t_*, (Model 1‴)

where ∆*INSE_TOTAL,t_* represents the difference in the use ratios of all major insecticides between years *t* and *t* − 1.

We also added the annual difference in insecticide use ratio as covariates into Model 2:

λ*_t_* = α + β_TEMP​_∆TEMP _t−1_ + β_NNFP​_∆INSE_NNFP,t_ + β_CART​_∆INSE_CART,t_
+ β_CARB​_∆INSE_CARB,t_ + ε_t_, (Model 2″)

λ*_t_* = α + β*_TEMP_*_​_∆*TEMP_t_*_−1_ + β*_TOTAL_*_​_∆*INSE_TOTAL,t_* + ε*_t_*, (Model 2‴)

where ∆*TEMP_t_*_−1_ is the difference in *TEMP* between year *t* − 1 and year *t* − 2 (∆*TEMP_t_*_−1_ = *TEMP_t_*_−1_ − *TEMP_t_*_−2_).

We conducted multiple linear regressions with these models for *S. frequens* and *S. infuscatum* as in the main analyses (see “Regression analyses” in Methods).

## S2.2. Results and interpretations

There was no significant effect of any insecticide use on *S. frequens* in the models (Table S3). Although the AIC value of Model 1″ (18.27) was smaller than that of Model 1 (22.07), the corresponding estimated coefficients of temperature were not much different. For *S. infuscatum*, the AIC value of Model 1 (12.63) was smaller than those of the models with the covariates of insecticide use (Model 1″, 14.13; Model 1‴, 12.71), and their estimated coefficients of temperature were similar (Table S4). Although in Models 2″ and 2‴ for *S. infuscatum* one covariate of insecticide use each was significant, their estimated coefficients of temperature were similar. These results reveal that insecticide use had no substantial influence on the results of this study.

**Table S3.** Results of regression analyses testing relation of summer temperature and insecticide use with population growth of *Sympetrum frequens*.

|  | Coefficients of explanatory variables (estimate ± SE) | | | | | |  |
| --- | --- | --- | --- | --- | --- | --- | --- |
|  | (Intercept) | ∆*TEMP_t_* | ∆*INSE*_NNFP,_*_t_* | *INSE*_CART,_*_t_* | ∆*INSE*_CARB,_*_t_* | ∆*INSE*_TOTAL,_*_t_* | AIC |
| Model 1 | −0.261  ± 0.170 | −0.232*  ± 0.089 | – | – | – | – | 22.07 |
| Model 1″ | −0.396  ± 0.186 | −0.318*  ± 0.106 | −0.002  ± 0.019 | −0.068  ± 0.038 | 0.029  ± 0.017 | – | 18.27 |
| Model 1‴ | −0.272  ± 0.181 | −0.218  ± 0.101 | – | – | – | 0.005  ± 0.013 | 23.89 |
|  | (Intercept) | ∆*TEMP_t–1_* | ∆*INSE*_NNFP,_*_t_* | *INSE*_CART,_*_t_* | ∆*INSE*_CARB,_*_t_* | ∆*INSE*_TOTAL_*_,t_* | AIC |
| Model 2 | −0.316  ± 0.206 | 0.129  ± 0.106 | – | – | – | – | 26.54 |
| Model 2″ | −0.276  ± 0.244 | 0.147  ± 0.103 | −0.010  ± 0.025 | −0.006  ± 0.039 | 0.051  ± 0.021 | – | 25.20 |
| Model 2‴ | −0.340  ± 0.201 | 0.142  ± 0.103 | – | – | – | 0.016  ± 0.013 | 26.66 |

NNFP: sum of neonicotinoids and fipronil; CART: cartap; CARB: carbosulfan; TOTAL: sum of all insecticides.

**P* < 0.05.

**Table S4.** Results of regression analyses testing relation of summer temperature and insecticide use with population growth of *Sympetrum infuscatum*.

|  | Coefficients of explanatory variables (estimate ± SE) | | | | | |  |
| --- | --- | --- | --- | --- | --- | --- | --- |
|  | (Intercept) | ∆*TEMP_t_* | ∆*INSE*_NNFP,_*_t_* | *INSE*_CART,_*_t_* | ∆*INSE*_CARB,_*_t_* | ∆*INSE*_TOTAL,_*_t_* | AIC |
| Model 1 | −0.254*  ± 0.110 | −0.242**  ± 0.058 | – | – | – | – | 12.63 |
| Model 1″ | −0.374  ± 0.153 | −0.279*  ± 0.088 | 0.014  ± 0.015 | −0.028  ± 0.031 | 0.012  ± 0.014 | – | 14.13 |
| Model 1‴ | −0.277*  ± 0.109 | −0.215**  ± 0.061 | – | – | – | 0.009  ± 0.008 | 12.71 |
|  | (Intercept) | ∆*TEMP_t–1_* | ∆*INSE*_NNFP,_*_t_* | *INSE*_CART,_*_t_* | ∆*INSE*_CARB,_*_t_* | ∆*INSE*_TOTAL,_*_t_* | AIC |
| Model 2 | −0.304*  ± 0.126 | 0.214**  ± 0.065 | – | – | – | – | 15.70 |
| Model 2″ | −0.333**  ± 0.079 | 0.238***  ± 0.034 | 0.016  ± 0.008 | 0.013  ± 0.013 | 0.032**  ± 0.007 | – | 0.43 |
| Model 2‴ | −0.335***  ± 0.065 | 0.230***  ± 0.033 | – | – | – | 0.022***  ± 0.004 | 1.84 |

NNFP: sum of neonicotinoids and fipronil; CART: cartap; CARB: carbosulfan; TOTAL: sum of all insecticides.

****P* < 0.001; ***P* < 0.01; **P* < 0.05.

# Note S3. Methods for calculation of summer habitat area

We defined the summer habitat area (SHA, km^2^) of *Sympetrum frequens* as the total area of regions where the mean temperature during July–August did not exceed 23 °C (Uéda, 1988). We obtained past temperature data for a 1-km^2^ grid from the NARO Agro-Meteorological Grid Square Data, a set of spatially interpolated data calculated from values measured by the Automated Meteorological Data Acquisition System of the Japan Meteorological Agency. We calculated SHA as the sum of the 1-km^2^ squares that met a range of 2-month mean temperature conditions (mean daily temperature during July–August did not exceed 21, 22, 23, 24 or 25 °C in Toyama Prefecture from 1981 to 2017).

# Note S4. Methods for projection of future population density

We projected the future population densities of *S. frequens* and *S. infuscatum* in Toyama by using each of the values of β of Model 1. Note that our intention was not to predict realistic population dynamics, but to perform a “what if” simulation under the assumption that only temperature can affect population dynamics, which provides insight into the potential effects of temperature itself. We used the same projection model as used in the projection of the past population density to calculate future population densities (see “Projection of population densities by using regression parameter” in Methods). We treated the intercept (α, a constant time trend independent of temperature) and error term (ε*_t_*) as 0 in the model, and calculated the annual population growth rate of the dragonfly (λ*_t_*) in year *t* with β as:

λ*_t_* = ln*N_t_* − ln*N_t_*_−1_ = β∆*TEMP_t_*

where *N_t_* (*N_t−1_*) is population density in year *t* (year *t* − 1), and ∆*TEMP_t_* is the difference in *TEMP* between year *t* and year *t* − 1. We set the population density of the first year (2019) at 1, and calculated abundance relative to the initial value in subsequent years during 2019 to 2100 in Toyama with the temperature prediction data from a global climate model. We forecast the future values of *TEMP* from 2019 to 2100 by using the MRI-CGCM3 global climate model, developed by the Meteorological Research Institute, JMA, under the RCP 8.5 and RCP 2.6 scenarios (i.e., 2.6–4.8 °C and 0.3–1.7 °C, respectively, rise in global mean temperature by 2100 relative to 1986–2005 average). Downscaled MRI-CGCM3 data were provided by the Social Implementation Program on Climate Change Adaptation Technology. Statistical bias was corrected by the scaling method using a normal probability distribution (Haerter et al., 2011). Minor bias was corrected using differences between the AMGSD and MRI-CGCM3 data in the 20-year (1981–2000) mean of daily mean temperature during July–August.

**
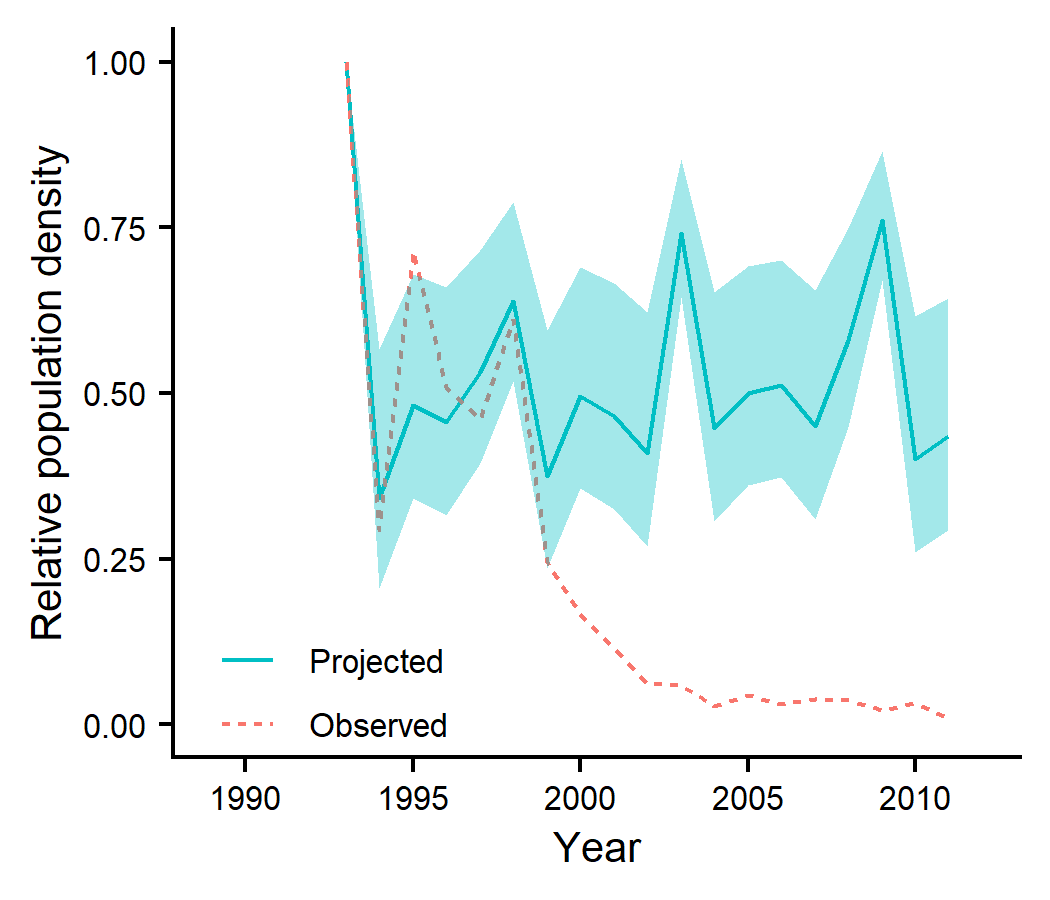
**

**Fig. S1** Relative population densities of *Sympetrum infuscatum* projected by using the estimated value of β of the regression analysis (Model 1) and the AMGSD temperature data (—), and the observed relative abundance (‑ ‑ ‑) in Toyama Prefecture. The shaded zone represents the range of projected relative population densities calculated by using the upper and lower limits of the 95% confidence interval of β. The observed data come from Futahashi (2012).

**
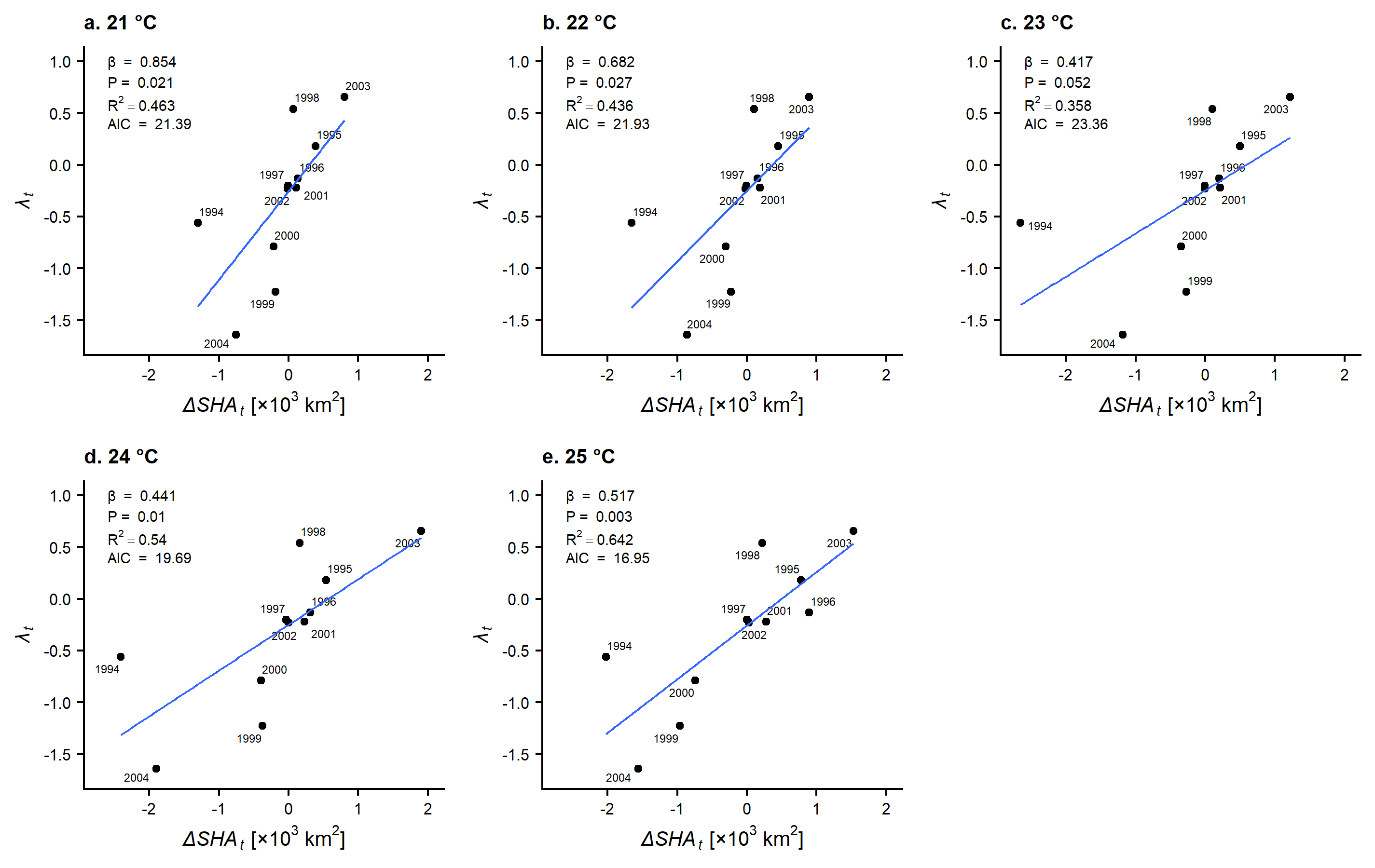
Fig. S2** Correlation between the difference in summer habitat area ($\Delta{SHA}_{t}$) and population growth rate of *Sympetrum frequens* ($\lambda_{t}$) in Toyama Prefecture from 1993 to 2004 under the different temperature thresholds for SHA (Model 1). The labels indicate year *t*. The results of regression analyses are shown in each panel.


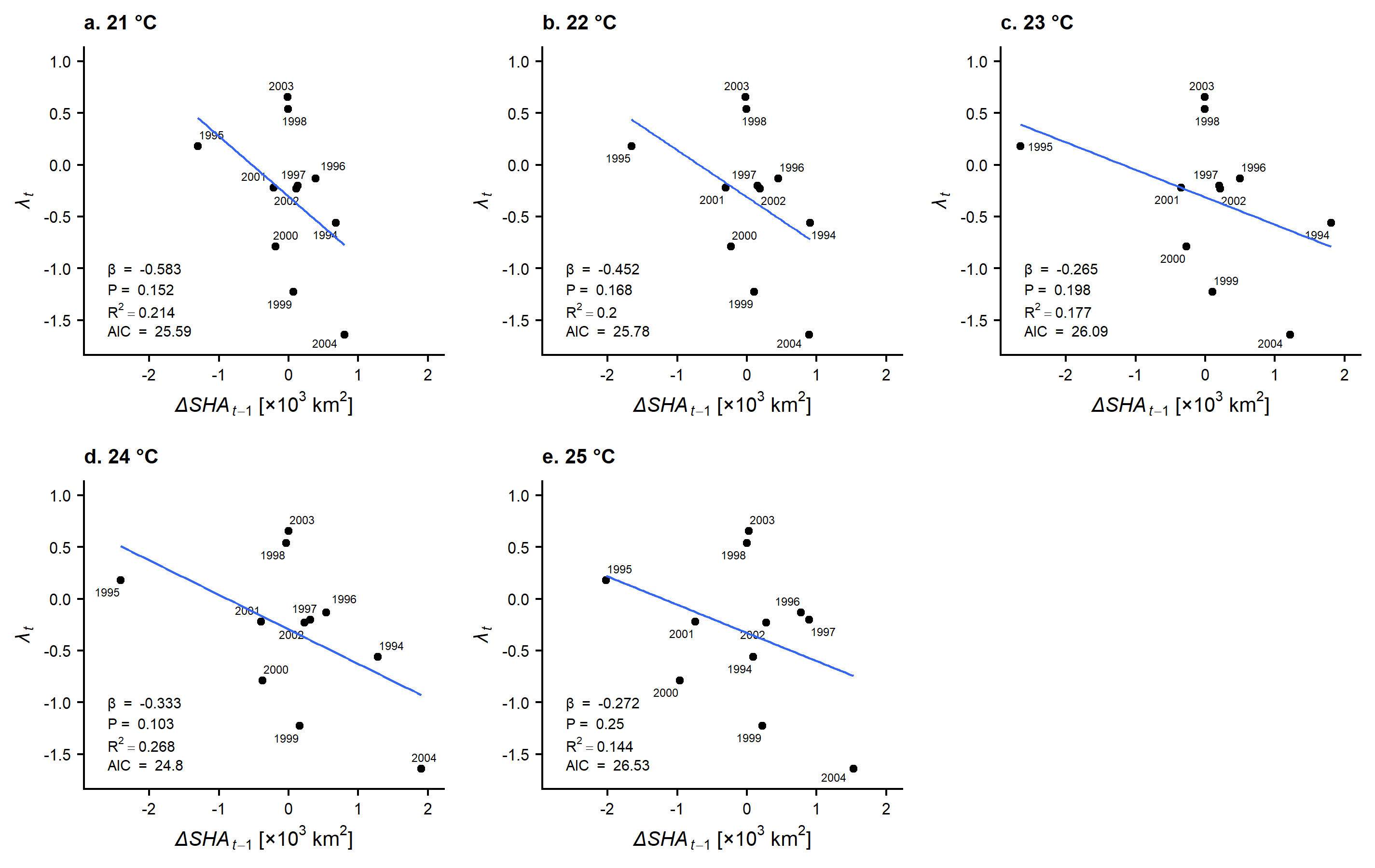


**Fig. S3** Correlation between the difference in summer habitat area ($\Delta{SHA}_{t-1}$) and population growth rate of *Sympetrum frequens* ($\lambda_{t}$) in Toyama Prefecture from 1993 to 2004 under the different temperature thresholds for SHA (Model 2). The labels indicate year *t*. The results of regression analyses are shown in each panel.


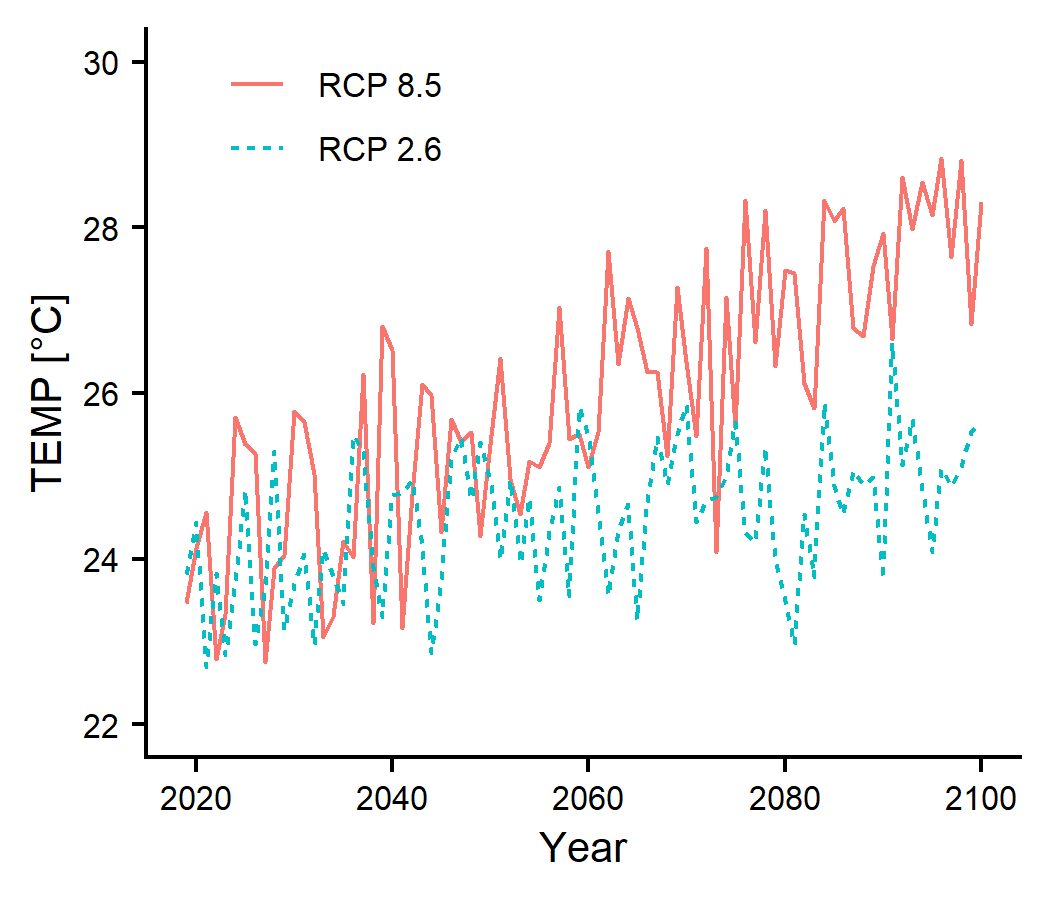


**Fig. S4** Forecast 90th percentile values of daily mean temperature during July–August (*TEMP*) calculated from the MRI-CGCM3 climate model under the RCP 8.5 (—) and RCP 2.6 (‑ ‑ ‑) scenarios in Toyama Prefecture.

**
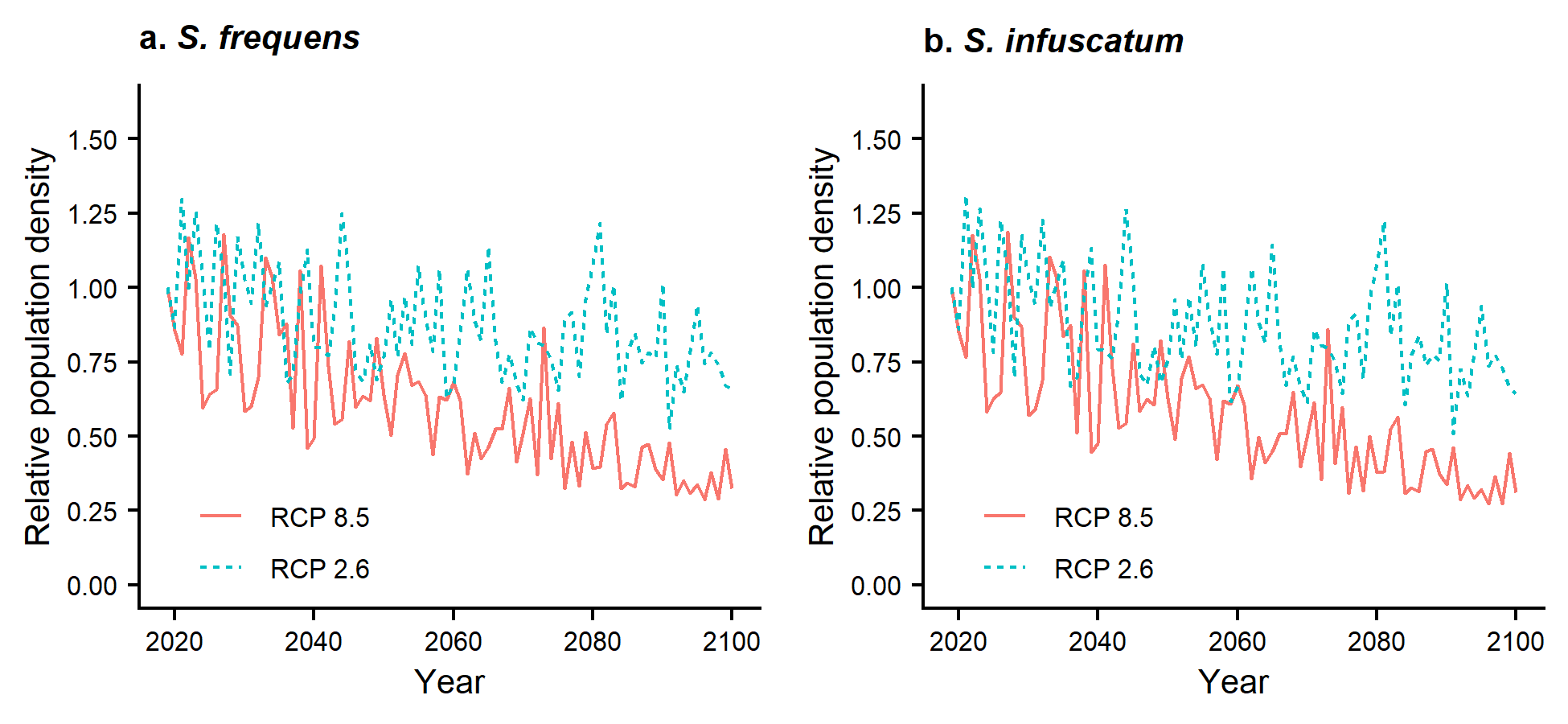
**

**Fig. S5** Forecast relative population densities of (a) *Sympetrum frequens* and (b) *S. infuscatum* projected by using each of the estimated values of β of the regression analysis (Model 1) and the 90th percentile values of daily mean temperature during July–August (*TEMP*) calculated from the MRI-CGCM3 climate model under the RCP 8.5 (—) and RCP 2.6 (‑ ‑ ‑) scenarios in Toyama Prefecture.

# References

Futahashi, R. Recent decline of red dragonflies in Toyama Prefecture. Nat. Insects **47**, 10–15 (2012).

Haerter, J. O., Hagemann, S., Moseley, C. & Piani, C. Climate model bias correction and the role of timescales. Hydrol. Earth Syst. Sci. **15**, 1065–1079 (2011).

Nakanishi, K., Yokomizo, H. & Hayashi, T. I. Were the sharp declines of dragonfly populations in the 1990s in Japan caused by fipronil and imidacloprid? An analysis of Hill’s causality for the case of *Sympetrum frequens*. Environ. Sci. Pollut. Res. **25**, 35352–35364 (2018).

Nakanishi, K., Uéda, T., Yokomizo, H. & Hayashi, T. I. Effects of systemic insecticides on the population dynamics of the dragonfly *Sympetrum frequens* in Japan: Statistical analyses using field census data from 2009 to 2016. Sci. Total Environ. **703**, 134499 (2020).

Uéda, T. Diversity in life history of the dragonfly *Sympetrum frequens* (Odonata: Insecta). Bull. Ishikawa Agric. Coll. **18**, 98–110 (1988).
