# Supplementary material for: Investigating effect of climate warming on the population declines of Sympetrum frequens during the 1990s in three regions in Japan
Source: Sci Rep. 2020 Jul 29;10:12719. doi: 10.1038/s41598-020-69532-8 (PMC7391746; doi:10.1038/s41598-020-69532-8)
Supplement: Supplementary file 2 — Supplementary Information 2. [file 41598_2020_69532_MOESM2_ESM.html]

Supplementary code


# Supplementary code

**Title**: Investigating effect of climate warming on the population declines of *Sympetrum frequens* during the 1990s in three regions in Japan

**Authors**: Kosuke Nakanishi, Dai Koide, Hiroyuki Yokomizo, Taku Kadoya, Takehiko I. Hayashi  
National Institute for Environmental Studies, Onogawa 16-2, Tsukuba, Ibaraki 305-8506, Japan  
nakanishi.kosuke@nies.go.jp (Nakanishi, K.)

## Package loading

```
require(dplyr)
```

```
## Warning: package 'dplyr' was built under R version 3.6.3
```

```
require(lmtest)
```

```
## Warning: package 'zoo' was built under R version 3.6.3
```

## 1. Data processing

```
d <- read.csv("Suppl_data.csv", header = TRUE) %>% 
  filter(
    pref == "Toyama" &
    year <= 2004
  ) %>%
  mutate(
    temp_1 = lag(temp),
    temp_diff = temp - lag(temp),
    temp_diff_1 = lag(temp_diff),
    total_diff = (fipronil + imidacloprid + dinotefuran + clothianidin + thiamethoxam + cartap + benfuracarb + carbosulfan + chlorantraniliprole) - lag(fipronil + imidacloprid + dinotefuran + clothianidin + thiamethoxam + cartap + benfuracarb + carbosulfan + chlorantraniliprole),
    nnfp_diff = (fipronil + imidacloprid + dinotefuran + clothianidin + thiamethoxam) - lag(fipronil + imidacloprid + dinotefuran + clothianidin + thiamethoxam),
    cart_diff = cartap - lag(cartap),
    carb_diff = carbosulfan - lag(carbosulfan),
    frequens_growth = log(frequens/lag(frequens)),
    infuscatum_growth = log(infuscatum/lag(infuscatum))
  )
```

## 2. Main analyses

### 2.1. *Sympetrum frequens*

```
# Null model
model_f_n <- lm(frequens_growth ~ 1, d)
summary(model_f_n)
```

```
## 
## Call:
## lm(formula = frequens_growth ~ 1, data = d)
## 
## Residuals:
##     Min      1Q  Median      3Q     Max 
## -1.3125 -0.3453  0.1084  0.3555  0.9859 
## 
## Coefficients:
##             Estimate Std. Error t value Pr(>|t|)
## (Intercept)  -0.3307     0.2102  -1.573    0.147
## 
## Residual standard error: 0.6973 on 10 degrees of freedom
##   (13 observations deleted due to missingness)
```

```
AIC(model_f_n)
```

```
## [1] 26.23653
```

```
# Model 1
model_1_f <- lm(frequens_growth ~ temp_diff, d)
summary(model_1_f)
```

```
## 
## Call:
## lm(formula = frequens_growth ~ temp_diff, data = d)
## 
## Residuals:
##     Min      1Q  Median      3Q     Max 
## -0.8984 -0.2727  0.1111  0.2639  0.7288 
## 
## Coefficients:
##             Estimate Std. Error t value Pr(>|t|)  
## (Intercept) -0.26061    0.16963  -1.536   0.1588  
## temp_diff   -0.23184    0.08917  -2.600   0.0287 *
## ---
## Signif. codes:  0 '***' 0.001 '**' 0.01 '*' 0.05 '.' 0.1 ' ' 1
## 
## Residual standard error: 0.5555 on 9 degrees of freedom
##   (13 observations deleted due to missingness)
## Multiple R-squared:  0.4289, Adjusted R-squared:  0.3655 
## F-statistic:  6.76 on 1 and 9 DF,  p-value: 0.02874
```

```
dwtest(model_1_f)
```

```
## 
##  Durbin-Watson test
## 
## data:  model_1_f
## DW = 1.6724, p-value = 0.3075
## alternative hypothesis: true autocorrelation is greater than 0
```

```
AIC(model_1_f)
```

```
## [1] 22.07391
```

```
# Model 2
model_2_f <- lm(frequens_growth ~ temp_diff_1, d)
summary(model_2_f)
```

```
## 
## Call:
## lm(formula = frequens_growth ~ temp_diff_1, data = d)
## 
## Residuals:
##      Min       1Q   Median       3Q      Max 
## -1.00877 -0.41881  0.07276  0.30666  0.93743 
## 
## Coefficients:
##             Estimate Std. Error t value Pr(>|t|)
## (Intercept)  -0.3163     0.2056  -1.539    0.158
## temp_diff_1   0.1294     0.1058   1.223    0.252
## 
## Residual standard error: 0.6806 on 9 degrees of freedom
##   (13 observations deleted due to missingness)
## Multiple R-squared:  0.1426, Adjusted R-squared:  0.0473 
## F-statistic: 1.497 on 1 and 9 DF,  p-value: 0.2523
```

```
dwtest(model_2_f)
```

```
## 
##  Durbin-Watson test
## 
## data:  model_2_f
## DW = 2.2867, p-value = 0.7429
## alternative hypothesis: true autocorrelation is greater than 0
```

```
AIC(model_2_f)
```

```
## [1] 26.54454
```

### 2.2. *Sympetrum infuscatum*

```
# Null model
model_i_n <- lm(infuscatum_growth ~ 1, d)
summary(model_i_n)
```

```
## 
## Call:
## lm(formula = infuscatum_growth ~ 1, data = d)
## 
## Residuals:
##      Min       1Q   Median       3Q      Max 
## -0.90117 -0.37312 -0.03921  0.25481  1.22037 
## 
## Coefficients:
##             Estimate Std. Error t value Pr(>|t|)  
## (Intercept)  -0.3276     0.1770  -1.851    0.094 .
## ---
## Signif. codes:  0 '***' 0.001 '**' 0.01 '*' 0.05 '.' 0.1 ' ' 1
## 
## Residual standard error: 0.5871 on 10 degrees of freedom
##   (13 observations deleted due to missingness)
```

```
AIC(model_i_n)
```

```
## [1] 22.45377
```

```
# Model 1
model_1_i <- lm(infuscatum_growth ~ temp_diff, d)
summary(model_1_i)
```

```
## 
## Call:
## lm(formula = infuscatum_growth ~ temp_diff, data = d)
## 
## Residuals:
##      Min       1Q   Median       3Q      Max 
## -0.40579 -0.19200 -0.02871  0.05106  0.80230 
## 
## Coefficients:
##             Estimate Std. Error t value Pr(>|t|)   
## (Intercept) -0.25448    0.11042  -2.305  0.04664 * 
## temp_diff   -0.24192    0.05805  -4.168  0.00242 **
## ---
## Signif. codes:  0 '***' 0.001 '**' 0.01 '*' 0.05 '.' 0.1 ' ' 1
## 
## Residual standard error: 0.3616 on 9 degrees of freedom
##   (13 observations deleted due to missingness)
## Multiple R-squared:  0.6587, Adjusted R-squared:  0.6208 
## F-statistic: 17.37 on 1 and 9 DF,  p-value: 0.00242
```

```
dwtest(model_1_i)
```

```
## 
##  Durbin-Watson test
## 
## data:  model_1_i
## DW = 1.6565, p-value = 0.2977
## alternative hypothesis: true autocorrelation is greater than 0
```

```
AIC(model_1_i)
```

```
## [1] 12.62869
```

```
# Model 2
model_2_i <- lm(infuscatum_growth ~ temp_diff_1, d)
summary(model_2_i)
```

```
## 
## Call:
## lm(formula = infuscatum_growth ~ temp_diff_1, data = d)
## 
## Residuals:
##     Min      1Q  Median      3Q     Max 
## -0.5488 -0.3938  0.1457  0.2155  0.7227 
## 
## Coefficients:
##             Estimate Std. Error t value Pr(>|t|)   
## (Intercept) -0.30378    0.12555  -2.420   0.0386 * 
## temp_diff_1  0.21385    0.06463   3.309   0.0091 **
## ---
## Signif. codes:  0 '***' 0.001 '**' 0.01 '*' 0.05 '.' 0.1 ' ' 1
## 
## Residual standard error: 0.4157 on 9 degrees of freedom
##   (13 observations deleted due to missingness)
## Multiple R-squared:  0.5488, Adjusted R-squared:  0.4987 
## F-statistic: 10.95 on 1 and 9 DF,  p-value: 0.009102
```

```
dwtest(model_2_i)
```

```
## 
##  Durbin-Watson test
## 
## data:  model_2_i
## DW = 2.123, p-value = 0.6465
## alternative hypothesis: true autocorrelation is greater than 0
```

```
AIC(model_2_i)
```

```
## [1] 15.69895
```

## 3. Supplementary analyses

### 3.1. Using absolute temperature (TEMP) (see Supp. Note S1)

#### 3.1.1. *Sympetrum frequens*

```
# Model 1
model_1_f_abs <- lm(frequens_growth ~ temp, d)
summary(model_1_f_abs)
```

```
## 
## Call:
## lm(formula = frequens_growth ~ temp, data = d)
## 
## Residuals:
##     Min      1Q  Median      3Q     Max 
## -1.1726 -0.2396  0.1680  0.3631  0.4955 
## 
## Coefficients:
##             Estimate Std. Error t value Pr(>|t|)  
## (Intercept)  11.9854     4.5200   2.652   0.0264 *
## temp         -0.5101     0.1871  -2.727   0.0234 *
## ---
## Signif. codes:  0 '***' 0.001 '**' 0.01 '*' 0.05 '.' 0.1 ' ' 1
## 
## Residual standard error: 0.5439 on 9 degrees of freedom
##   (13 observations deleted due to missingness)
## Multiple R-squared:  0.4524, Adjusted R-squared:  0.3915 
## F-statistic: 7.434 on 1 and 9 DF,  p-value: 0.02335
```

```
dwtest(model_1_f_abs)
```

```
## 
##  Durbin-Watson test
## 
## data:  model_1_f_abs
## DW = 1.1151, p-value = 0.05805
## alternative hypothesis: true autocorrelation is greater than 0
```

```
AIC(model_1_f_abs)
```

```
## [1] 21.61287
```

```
# Model 2
model_2_f_abs <- lm(frequens_growth ~ temp_1, d)
summary(model_2_f_abs)
```

```
## 
## Call:
## lm(formula = frequens_growth ~ temp_1, data = d)
## 
## Residuals:
##      Min       1Q   Median       3Q      Max 
## -0.88793 -0.33242  0.03552  0.33174  0.90790 
## 
## Coefficients:
##             Estimate Std. Error t value Pr(>|t|)  
## (Intercept)  -7.0254     3.6696  -1.914   0.0878 .
## temp_1        0.2808     0.1537   1.827   0.1010  
## ---
## Signif. codes:  0 '***' 0.001 '**' 0.01 '*' 0.05 '.' 0.1 ' ' 1
## 
## Residual standard error: 0.6278 on 9 degrees of freedom
##   (13 observations deleted due to missingness)
## Multiple R-squared:  0.2705, Adjusted R-squared:  0.1894 
## F-statistic: 3.337 on 1 and 9 DF,  p-value: 0.101
```

```
dwtest(model_2_f_abs)
```

```
## 
##  Durbin-Watson test
## 
## data:  model_2_f_abs
## DW = 2.1181, p-value = 0.5867
## alternative hypothesis: true autocorrelation is greater than 0
```

```
AIC(model_2_f_abs)
```

```
## [1] 24.76718
```

#### 3.1.2. *Sympetrum infuscatum*

```
# Model 1
model_1_i_abs <- lm(infuscatum_growth ~ temp, d)
summary(model_1_i_abs)
```

```
## 
## Call:
## lm(formula = infuscatum_growth ~ temp, data = d)
## 
## Residuals:
##      Min       1Q   Median       3Q      Max 
## -0.48094 -0.24081 -0.03774  0.05750  1.20691 
## 
## Coefficients:
##             Estimate Std. Error t value Pr(>|t|)  
## (Intercept)   9.8281     3.8698   2.540   0.0317 *
## temp         -0.4206     0.1602  -2.626   0.0275 *
## ---
## Signif. codes:  0 '***' 0.001 '**' 0.01 '*' 0.05 '.' 0.1 ' ' 1
## 
## Residual standard error: 0.4657 on 9 degrees of freedom
##   (13 observations deleted due to missingness)
## Multiple R-squared:  0.4338, Adjusted R-squared:  0.3709 
## F-statistic: 6.896 on 1 and 9 DF,  p-value: 0.02754
```

```
dwtest(model_1_i_abs)
```

```
## 
##  Durbin-Watson test
## 
## data:  model_1_i_abs
## DW = 2.0113, p-value = 0.5455
## alternative hypothesis: true autocorrelation is greater than 0
```

```
AIC(model_1_i_abs)
```

```
## [1] 18.19642
```

```
# Model 2
model_2_i_abs <- lm(infuscatum_growth ~ temp_1, d)
summary(model_2_i_abs)
```

```
## 
## Call:
## lm(formula = infuscatum_growth ~ temp_1, data = d)
## 
## Residuals:
##      Min       1Q   Median       3Q      Max 
## -0.51099 -0.19130 -0.04846  0.07519  0.65843 
## 
## Coefficients:
##             Estimate Std. Error t value Pr(>|t|)   
## (Intercept) -8.66233    2.31271  -3.746  0.00459 **
## temp_1       0.34959    0.09687   3.609  0.00567 **
## ---
## Signif. codes:  0 '***' 0.001 '**' 0.01 '*' 0.05 '.' 0.1 ' ' 1
## 
## Residual standard error: 0.3957 on 9 degrees of freedom
##   (13 observations deleted due to missingness)
## Multiple R-squared:  0.5913, Adjusted R-squared:  0.5459 
## F-statistic: 13.02 on 1 and 9 DF,  p-value: 0.005671
```

```
dwtest(model_2_i_abs)
```

```
## 
##  Durbin-Watson test
## 
## data:  model_2_i_abs
## DW = 1.896, p-value = 0.438
## alternative hypothesis: true autocorrelation is greater than 0
```

```
AIC(model_2_i_abs)
```

```
## [1] 14.61046
```

### 3.2. Including insecticides use (TEMP) (see Supp. Note S2)

#### 3.2.1. *Sympetrum frequens*

```
# Model 1
## Individual insecticide
model_1_f_nnfp <- lm(frequens_growth ~ temp_diff + nnfp_diff + cart_diff + carb_diff, d)
summary(model_1_f_nnfp)
```

```
## 
## Call:
## lm(formula = frequens_growth ~ temp_diff + nnfp_diff + cart_diff + 
##     carb_diff, data = d)
## 
## Residuals:
##      Min       1Q   Median       3Q      Max 
## -0.64266 -0.06168  0.06593  0.24074  0.41312 
## 
## Coefficients:
##              Estimate Std. Error t value Pr(>|t|)  
## (Intercept) -0.396095   0.185770  -2.132   0.0770 .
## temp_diff   -0.318159   0.105778  -3.008   0.0238 *
## nnfp_diff   -0.001873   0.018563  -0.101   0.9229  
## cart_diff   -0.068103   0.037949  -1.795   0.1229  
## carb_diff    0.029053   0.016958   1.713   0.1375  
## ---
## Signif. codes:  0 '***' 0.001 '**' 0.01 '*' 0.05 '.' 0.1 ' ' 1
## 
## Residual standard error: 0.4356 on 6 degrees of freedom
##   (13 observations deleted due to missingness)
## Multiple R-squared:  0.7658, Adjusted R-squared:  0.6097 
## F-statistic: 4.906 on 4 and 6 DF,  p-value: 0.04234
```

```
dwtest(model_1_f_nnfp)
```

```
## 
##  Durbin-Watson test
## 
## data:  model_1_f_nnfp
## DW = 2.1186, p-value = 0.7227
## alternative hypothesis: true autocorrelation is greater than 0
```

```
AIC(model_1_f_nnfp)
```

```
## [1] 18.26743
```

```
## Total insecticides
model_1_f_total <- lm(frequens_growth ~ temp_diff + total_diff, d)
summary(model_1_f_total)
```

```
## 
## Call:
## lm(formula = frequens_growth ~ temp_diff + total_diff, data = d)
## 
## Residuals:
##     Min      1Q  Median      3Q     Max 
## -0.9565 -0.2895  0.1345  0.3160  0.7610 
## 
## Coefficients:
##              Estimate Std. Error t value Pr(>|t|)  
## (Intercept) -0.271792   0.180941  -1.502   0.1715  
## temp_diff   -0.218259   0.100705  -2.167   0.0621 .
## total_diff   0.004635   0.012525   0.370   0.7209  
## ---
## Signif. codes:  0 '***' 0.001 '**' 0.01 '*' 0.05 '.' 0.1 ' ' 1
## 
## Residual standard error: 0.5842 on 8 degrees of freedom
##   (13 observations deleted due to missingness)
## Multiple R-squared:  0.4385, Adjusted R-squared:  0.2982 
## F-statistic: 3.124 on 2 and 8 DF,  p-value: 0.09938
```

```
dwtest(model_1_f_total)
```

```
## 
##  Durbin-Watson test
## 
## data:  model_1_f_total
## DW = 1.5628, p-value = 0.2539
## alternative hypothesis: true autocorrelation is greater than 0
```

```
AIC(model_1_f_total)
```

```
## [1] 23.88719
```

```
# Model 2
## Individual insecticide
model_2_f_nnfp <- lm(frequens_growth ~ temp_diff_1 + nnfp_diff + cart_diff + carb_diff, d)
summary(model_2_f_nnfp)
```

```
## 
## Call:
## lm(formula = frequens_growth ~ temp_diff_1 + nnfp_diff + cart_diff + 
##     carb_diff, data = d)
## 
## Residuals:
##     Min      1Q  Median      3Q     Max 
## -0.8957 -0.1133  0.1043  0.1902  0.9083 
## 
## Coefficients:
##              Estimate Std. Error t value Pr(>|t|)  
## (Intercept) -0.275797   0.244251  -1.129   0.3019  
## temp_diff_1  0.146671   0.103417   1.418   0.2059  
## nnfp_diff   -0.010217   0.025151  -0.406   0.6987  
## cart_diff   -0.005565   0.038626  -0.144   0.8902  
## carb_diff    0.050724   0.021309   2.380   0.0547 .
## ---
## Signif. codes:  0 '***' 0.001 '**' 0.01 '*' 0.05 '.' 0.1 ' ' 1
## 
## Residual standard error: 0.597 on 6 degrees of freedom
##   (13 observations deleted due to missingness)
## Multiple R-squared:  0.5602, Adjusted R-squared:  0.267 
## F-statistic: 1.911 on 4 and 6 DF,  p-value: 0.228
```

```
dwtest(model_2_f_nnfp)
```

```
## 
##  Durbin-Watson test
## 
## data:  model_2_f_nnfp
## DW = 2.4977, p-value = 0.9071
## alternative hypothesis: true autocorrelation is greater than 0
```

```
AIC(model_2_f_nnfp)
```

```
## [1] 25.2007
```

```
## Total insecticides
model_2_f_total <- lm(frequens_growth ~ temp_diff_1 + total_diff, d)
summary(model_2_f_total)
```

```
## 
## Call:
## lm(formula = frequens_growth ~ temp_diff_1 + total_diff, data = d)
## 
## Residuals:
##      Min       1Q   Median       3Q      Max 
## -1.09919 -0.34803  0.00974  0.42026  0.88222 
## 
## Coefficients:
##             Estimate Std. Error t value Pr(>|t|)
## (Intercept) -0.33975    0.20102  -1.690    0.129
## temp_diff_1  0.14152    0.10348   1.368    0.209
## total_diff   0.01626    0.01329   1.224    0.256
## 
## Residual standard error: 0.6626 on 8 degrees of freedom
##   (13 observations deleted due to missingness)
## Multiple R-squared:  0.2777, Adjusted R-squared:  0.09717 
## F-statistic: 1.538 on 2 and 8 DF,  p-value: 0.2721
```

```
dwtest(model_2_f_total)
```

```
## 
##  Durbin-Watson test
## 
## data:  model_2_f_total
## DW = 2.0388, p-value = 0.6222
## alternative hypothesis: true autocorrelation is greater than 0
```

```
AIC(model_2_f_total)
```

```
## [1] 26.65751
```

#### 3.2.2. *Sympetrum infuscatum*

```
# Model 1
## Individual insecticide
model_1_i_nnfp <- lm(infuscatum_growth ~ temp_diff + nnfp_diff + cart_diff + carb_diff, d)
summary(model_1_i_nnfp)
```

```
## 
## Call:
## lm(formula = infuscatum_growth ~ temp_diff + nnfp_diff + cart_diff + 
##     carb_diff, data = d)
## 
## Residuals:
##      Min       1Q   Median       3Q      Max 
## -0.43970 -0.11342 -0.03441  0.04592  0.70690 
## 
## Coefficients:
##             Estimate Std. Error t value Pr(>|t|)  
## (Intercept) -0.37421    0.15392  -2.431   0.0511 .
## temp_diff   -0.27945    0.08764  -3.188   0.0189 *
## nnfp_diff    0.01412    0.01538   0.918   0.3939  
## cart_diff   -0.02784    0.03144  -0.885   0.4101  
## carb_diff    0.01159    0.01405   0.825   0.4412  
## ---
## Signif. codes:  0 '***' 0.001 '**' 0.01 '*' 0.05 '.' 0.1 ' ' 1
## 
## Residual standard error: 0.3609 on 6 degrees of freedom
##   (13 observations deleted due to missingness)
## Multiple R-squared:  0.7733, Adjusted R-squared:  0.6221 
## F-statistic: 5.116 on 4 and 6 DF,  p-value: 0.0387
```

```
dwtest(model_1_i_nnfp)
```

```
## 
##  Durbin-Watson test
## 
## data:  model_1_i_nnfp
## DW = 2.4311, p-value = 0.8942
## alternative hypothesis: true autocorrelation is greater than 0
```

```
AIC(model_1_i_nnfp)
```

```
## [1] 14.1302
```

```
## Total insecticies
model_1_i_total <- lm(infuscatum_growth ~ temp_diff + total_diff, d)
summary(model_1_i_total)
```

```
## 
## Call:
## lm(formula = infuscatum_growth ~ temp_diff + total_diff, data = d)
## 
## Residuals:
##      Min       1Q   Median       3Q      Max 
## -0.31789 -0.15543 -0.09187  0.04899  0.84916 
## 
## Coefficients:
##              Estimate Std. Error t value Pr(>|t|)   
## (Intercept) -0.276944   0.108853  -2.544  0.03448 * 
## temp_diff   -0.214638   0.060583  -3.543  0.00759 **
## total_diff   0.009311   0.007535   1.236  0.25164   
## ---
## Signif. codes:  0 '***' 0.001 '**' 0.01 '*' 0.05 '.' 0.1 ' ' 1
## 
## Residual standard error: 0.3514 on 8 degrees of freedom
##   (13 observations deleted due to missingness)
## Multiple R-squared:  0.7134, Adjusted R-squared:  0.6418 
## F-statistic: 9.957 on 2 and 8 DF,  p-value: 0.006747
```

```
dwtest(model_1_i_total)
```

```
## 
##  Durbin-Watson test
## 
## data:  model_1_i_total
## DW = 1.4429, p-value = 0.1897
## alternative hypothesis: true autocorrelation is greater than 0
```

```
AIC(model_1_i_total)
```

```
## [1] 12.7073
```

```
# Model 2
## Individual insecticide
model_2_i_nnfp <- lm(infuscatum_growth ~ temp_diff_1 + nnfp_diff + cart_diff + carb_diff, d)
summary(model_2_i_nnfp)
```

```
## 
## Call:
## lm(formula = infuscatum_growth ~ temp_diff_1 + nnfp_diff + cart_diff + 
##     carb_diff, data = d)
## 
## Residuals:
##      Min       1Q   Median       3Q      Max 
## -0.27663 -0.07324 -0.02141  0.06916  0.30498 
## 
## Coefficients:
##              Estimate Std. Error t value Pr(>|t|)    
## (Intercept) -0.332529   0.079206  -4.198 0.005697 ** 
## temp_diff_1  0.237595   0.033536   7.085 0.000397 ***
## nnfp_diff    0.015639   0.008156   1.917 0.103630    
## cart_diff    0.012399   0.012526   0.990 0.360451    
## carb_diff    0.031528   0.006910   4.563 0.003841 ** 
## ---
## Signif. codes:  0 '***' 0.001 '**' 0.01 '*' 0.05 '.' 0.1 ' ' 1
## 
## Residual standard error: 0.1936 on 6 degrees of freedom
##   (13 observations deleted due to missingness)
## Multiple R-squared:  0.9348, Adjusted R-squared:  0.8913 
## F-statistic:  21.5 on 4 and 6 DF,  p-value: 0.001056
```

```
dwtest(model_2_i_nnfp)
```

```
## 
##  Durbin-Watson test
## 
## data:  model_2_i_nnfp
## DW = 1.748, p-value = 0.3978
## alternative hypothesis: true autocorrelation is greater than 0
```

```
AIC(model_2_i_nnfp)
```

```
## [1] 0.4256414
```

```
## Total insecticides
model_2_i_total <- lm(infuscatum_growth ~ temp_diff_1 + total_diff, d)
summary(model_2_i_total)
```

```
## 
## Call:
## lm(formula = infuscatum_growth ~ temp_diff_1 + total_diff, data = d)
## 
## Residuals:
##      Min       1Q   Median       3Q      Max 
## -0.28397 -0.12942  0.05683  0.10685  0.35083 
## 
## Coefficients:
##              Estimate Std. Error t value Pr(>|t|)    
## (Intercept) -0.335349   0.065071  -5.154 0.000870 ***
## temp_diff_1  0.230080   0.033497   6.869 0.000129 ***
## total_diff   0.021857   0.004302   5.081 0.000952 ***
## ---
## Signif. codes:  0 '***' 0.001 '**' 0.01 '*' 0.05 '.' 0.1 ' ' 1
## 
## Residual standard error: 0.2145 on 8 degrees of freedom
##   (13 observations deleted due to missingness)
## Multiple R-squared:  0.8933, Adjusted R-squared:  0.8666 
## F-statistic: 33.47 on 2 and 8 DF,  p-value: 0.0001298
```

```
dwtest(model_2_i_total)
```

```
## 
##  Durbin-Watson test
## 
## data:  model_2_i_total
## DW = 1.432, p-value = 0.2168
## alternative hypothesis: true autocorrelation is greater than 0
```

```
AIC(model_2_i_total)
```

```
## [1] 1.843418
```
